# Variation of 46 Innate Immune Genes Evaluated for their Contribution in Pneumococcal Meningitis Susceptibility and Outcome

**DOI:** 10.1016/j.ebiom.2016.07.011

**Published:** 2016-07-12

**Authors:** Bart Ferwerda, Mercedes Valls Serón, Aldo Jongejan, Aeilko H. Zwinderman, Madelijn Geldhoff, Arie van der Ende, Frank Baas, Matthijs C. Brouwer, Diederik van de Beek

**Affiliations:** a Department of Neurology, Center of Infection and Immunity Amsterdam (CINIMA), Academic Medical Center, P.O. Box 22660, Amsterdam, The Netherlands; b Bioinformatics Laboratory, Academic Medical Center, P.O. Box 22660, Amsterdam, The Netherlands; c Department of Clinical Epidemiology, Biostatistics, and Bioinformatics, Academic Medical Center, University of Amsterdam, P.O. Box 22660, Amsterdam, The Netherlands; d Department of Medical Microbiology, Center of Infection and Immunity Amsterdam (CINIMA), Academic Medical Center, P.O. Box 22660, Amsterdam, The Netherlands; e The Netherlands Reference Laboratory for Bacterial Meningitis, Center of Infection and Immunity Amsterdam (CINIMA), Academic Medical Center, P.O. Box 22660, Amsterdam, The Netherlands; f Department of Clinical Genetics, Academic Medical Center, P.O. Box 22660, Amsterdam, The Netherlands

**Keywords:** CSF, cerebrospinal fluid, FDR, False Discovery Rate, GATK, genome analysis tool kit, GOS, Glasgow Outcome Scale, HWE, Hardy–Weinberg equilibrium, IPD, invasive pneumococcal disease, LTA, lipoteichoic acid, MAF, minor allele frequency, MDS, multidimensional scaling, NLRs, NOD-like receptors, NRLBM, Netherlands Reference Laboratories For Bacterial Meningitis, OR, odds ratio, PAMPS, pathogen-associated molecular pattern molecules, PCR, polymerase chain reaction, RVIS, residual variation intolerance score, SKAT, SNP-set (Sequence) Kernel Association Test, TLR, Toll-like receptors, Innate immunity, Pneumococcal meningitis, IRAK4, NOD2

## Abstract

Pneumococcal meningitis is the most common and severe form of bacterial meningitis. Early recognition of the pathogen and subsequent innate immune response play a vital role in disease susceptibility and outcome. Genetic variations in innate immune genes can alter the immune response and influence susceptibility and outcome of meningitis disease.

Here we conducted a sequencing study of coding regions from 46 innate immune genes in 435 pneumococcal meningitis patients and 416 controls, to determine the role of genetic variation on pneumococcal meningitis susceptibility and disease outcome.

Strongest signals for susceptibility were rs56078309 *CXCL1* (p = 4.8e − 04) and rs2008521 in *CARD8* (p = 6.1e − 04). For meningitis outcome the rs2067085 in *NOD2* (p = 5.1e − 04) and rs4251552 of *IRAK4* were the strongest associations with unfavorable outcome (p = 6.7e − 04). Haplotype analysis showed a haplotype block, determined by *IRAK4* rs4251552, significantly associated with unfavorable outcome (p = 0.004). Cytokine measurements from cerebrospinal fluid showed that with the *IRAK4* rs4251552 G risk allele had higher levels of IL-6 compared to individuals with A/A genotype (p = 0.04).

We show that genetic variation within exons and flanking regions of 46 innate immunity genes does not yield significant association with pneumococcal meningitis. The strongest identified signal IRAK4 does imply a potential role of genetic variation in pneumococcal meningitis.

## Highlights

• Exome variations of 46 innate immune genes show no association with pneumococcal meningitis susceptibility and outcome.
• Variations that have previously been associated with invasive pneumococcal disease or meningitis could not be replicated.
• Highest signals found were variations in CXCL1 and CARD8 for susceptibility and NOD2 and IRAK4 for outcome.
• The *IRAK4* rs4251552 variant shows a trend where patients with a G-allele have higher IL-6 levels in their liquor.

The pneumococcus is the most common causative pathogen of bacterial meningitis. We investigated the association of genetic variation within the coding sequences and flanking intronic regions of 46 innate immunity genes. These genes are responsible for our first line of defense during invading pathogens. In a cohort of 435 pneumococcal meningitis patients and 416 controls we found that variation within *CXCL1* and *CARD8* were the strongest signals associated with pneumococcal meningitis susceptibility. For disease severity we identified variations in *NOD2* and *IRAK4* genes to be associated with outcome. These findings can help in the understanding and treatment of pneumococcal meningitis.

## 1 Introduction

Community-acquired meningitis is a life-threatening infection of the membranes surrounding the brain and spinal cord. Pneumococcal meningitis is the most common and severe form of bacterial meningitis. Fatality rates are substantial, and long-term sequelae develop in about half of survivors (Brouwer et al., 2010, Schut et al., 2012, van de Beek et al., 2012, van de Beek et al., 2004, Zoons et al., 2008). Vaccination has decreased the incidence of invasive pneumococcal disease in infants and recently also in the adult population (Bijlsma et al., 2015, McIntyre et al., 2012, Tsai et al., 2008).

*Streptococcus pneumoniae* is a human commensal strain adapted to colonize the nasopharynx (Brown et al., 2015). However, after asymptomatic colonization translocation of the pneumococcus to the respiratory tract, sinuses and nasal cavity, *S. pneumoniae* can cause pneumonia, acute sinusitis, otitis media, bacteremia, sepsis and meningitis (Brown et al., 2015, Mook-Kanamori et al., 2011, van de Beek et al., 2006). One of the first host determinants of developing an infection is the recognition and clearance of the pneumococcal strains with initiation of an inflammatory response by the innate immune response. The innate immune response depends on specific pathogen-associated molecular pattern molecules (PAMPS) of the pneumococcus. For example, peptidoglycan and lipoteichoic acid (LTA), are recognized by membrane surface and intracellular Toll-like receptors (TLRs) found on leukocyte cells (Santos-Sierra et al., 2006). After PAMP recognition, intracellular signaling initiates the activation of transcription factors. This leads to the induction of small cell signaling proteins, called cytokines, which are responsible for the inflammatory response and attracts immune cells to the site of infection (Akira et al., 2006). TLRs, like TLR2, TLR4, TLR9 and NOD-like receptors (NLRs) as NOD2 are known to be important in the recognition of invasive pneumococcal strains (Koppe et al., 2012). Differences within these or underlying signaling proteins, caused by genetic variations, can contribute to differences in the immune response affecting the susceptibility to disease and its severity.

Genetic variations *TLR4, TIRAP, NFKBIA and NFKBIB, TNF, IL10, IL-6, IRAK4* and *IKBKG* have been previously described to influence susceptibility to invasive pneumococcal disease (Brouwer et al., 2009, Chapman et al., 2007). The role of genetic variation in innate immune genes in pneumococcal meningitis and its effect on disease outcome is less well known. In this study, we sequenced the exons and flanking intron borders of all known genes that are important in innate immune signaling pathway in a pneumococcal meningitis patient population for which detailed clinical data has been collected. This gives us the opportunity to study association of innate immune gene variation with disease susceptibility and outcome, and to gain further insight in the role of genetic variation in the innate immune system during pneumococcal meningitis.

## 2 Material and Methods

### 2.1 Dutch Bacterial Meningitis Cohort

In a nationwide prospective cohort study (MeninGene) we included adult patients, age of 16 years and older, with community-acquired bacterial meningitis with positive CSF cultures who were identified by the Netherlands Reference Laboratories for Bacterial Meningitis (NRLBM). The cohort and inclusion procedure is described elsewhere (Bijlsma et al., 2016).

A total of 1300 patients and partners or non-related proxies, living in the same residence, used as controls included during 2006 and 2011, were included in this study. Patient data were collected in an online case record form and included presenting characteristics, treatment, complications and outcome. Patient outcome was graded at discharge according the Glasgow Outcome Scale (GOS) (Jennett et al., 1976). A score of one on this five point scale indicates death, score of two vegetative state, score three severe disability, score four moderate disability, and a score of five mild or no disability. We considered a score of 5 a favorable outcome and scores 1 to 4 were defined as unfavorable outcome.

Blood for DNA extraction was withdrawn from the patients and collected in sodium/EDTA tubes. Isolation of the DNA was performed with the Gentra Puregene isolation kit (Qiagen) according to manufacturer's protocol, thereafter the yield and quality of the extractions were determined to ensure appropriate conditions for genotyping.

### 2.2 Ethical Approval

This study was approved by the ethics committee of the Academic Medical Center, Amsterdam, the Netherlands. Informed consent was obtained from all participating individuals or legally authorized representatives. The study was conducted according to the principles of the Declaration of Helsinki (version of 2013. Fortaleza, Brazil) and in accordance with the Medical Research Involving Human Subjects Act (WMO) and other guidelines, regulations and acts.

### 2.3 Marker Selection

Forty-six innate immune genes were selected for exome sequencing (Supplementary Table 1). Inclusion criteria were: (1) genes with known involvement of pneumococcal recognition, (2) genes with variations that have previously been associated with invasive pneumococcal disease and meningitis and (3) genes that encode for related downstream signaling and transcription proteins in the TLR, NOD and inflammasome signaling pathways. This led to the inclusion of 46 innate immune genes (Supplement Table 1).

### 2.4 Solid Sequencing

DNA concentration was determined by means of fluorometric measurement (Qubit, Thermo) and quality was checked by means of determining the absence of degradation and presence of High Molecular weight DNA. Circa 1,5 μg DNA was sheared by sonication followed by barcoded adaptor ligated library construction, using the Biomek FX automated liquid handler (Beckman Coulter). Solid fragment Library preparation kit and solid barcoded adaptors were used according manufacturers protocol (Life Technologies, 5500 SOLID™ Fragment Library Core Kit, Catalog number 4464412). Each sample was generated using a separate 10 bp barcode incorporated in the adaptor sequence. After 8 cycles of amplification using the library prep kit supplied PCR primers, PCR mix and PCR protocol and purified twice-using ampure. Two rounds of hybridization capture was performed using a Custom Complement Capture (Nimblegen, pn 130204_HG19_CompCapV2_MJ_EZ_HX3). Equimolar pooling of the captured libraries was based on concentration and average sample size. Emulsion PCR was performed using the Solid EZ Bead Emulsifier and Amplifier (Applied Biosystems). Sequencing was performed on the Solid 5500xl sequencer (Life technologies) generating paired-end reads (50 bp forward and 35 bp reverse).

### 2.5 Alignment and Variation Calling Pipeline

Paired-end reads for each individual were merged using Picard (version 1.92) and aligned to the GRch37/HG19 reference genome using the Lifescope aligner (version 2.5.1, Applied Biosystems). To minimize mismatched bases between reads, realignment was performed using the RealignerTargetCreator function in GATK (version 2.7-4) using the intervals from Mills_and_1000G_gold_standard.indels.hg19.vcf. Mate information can be changed during realignment and changes were fixed using Picard. Samples were recalibrated with GATK Recalibrate and variants were called using GATKs HaplotypeCaller (version 3.3-0) with default settings and only adjusting the minimal mapping quality score to 30. Variants were then filtered on a minimal read depth of 20, allele balance between 0.2 and 0.8 for heterozygotes, > 0.9 for homozygotes and genotype quality of 99. After filtering, all samples were combined and genotyped by using GATK CombineGVCFs and GenotypeGVCFs (version 3.3-0) and converted to PLINK (Chang et al., 2015). Finally, all chromosomal locations for found variants were annotated using SnpEff and the UCSC variant annotation integrator tool (https://genome.ucsc.edu/) (Cingolani et al., 2012).

### 2.6 Variants and Sample QC

After alignment and variation calling, we removed all individuals of which we found that there was a third, or higher, degree of relatedness. Reported ancestry was used to exclude non-European ancestry to account for population stratification. Because the ancestry was reported we also evaluated the effect of ancestry for all common variants (MAF ≥ 0.01) by calculating the multidimensional scaling (MDS) analysis with PLINK and including them as covariates.

All individuals with missingness of 5% or heterozygosity above or below 3 standard deviations from the mean were removed. Finally, variations were removed from the analysis when having a missingness > 5% and HWE p-value < 1.0e − 05, QC steps were conducted with PLINK (Chang et al., 2015). Variations in short insertions and deletions (indels) are difficult to correctly detect with the short read sequencing methodologies and were therefore excluded from the association analysis (Ng et al., 2009).

### 2.7 Cytokines

Cytokines IL-6, TNF-α, IL1-β and IL-18 were determined in the CSF collected from the diagnostic lumbar puncture. All analytes were measured with Luminex® xMAP® technology using Milliplex® map multiplex assay's (Millipore, Billerica) as descripted by Koopmans et al. (Koopmans et al., 2014).

### 2.8 Residual Variation Intolerance Score (RVIS)

To evaluate if genes had more functional variation then expected based on the neutral variations of that gene we calculated the RVIS of each gene as described by Petrovski et al. (Petrovski et al., 2013). RVIS calculations were performed with R (R, 2008).

### 2.9 Statistics

PLINK was used for the single variation analysis (Chang et al., 2015). We performed the Fisher exact-test for detecting allelic associations between groups. Variations were considered significant when the False Discovery Rate (FDR) adjusted p-value for the total number of tests was below 0.05. We tested 1854 and 1385 variations for susceptibility and outcome, respectively therefore p-values below 2.7e − 05 and 3.6e − 05 were statistically significant.

For all MAF higher or equal to a frequency of 0.01% we reran the analysis with a logistic regression including the first ten MDS, to correct for possible population stratification, age and if the patients were immunosuppressed as covariates.

To study haplotype association, we used Haploview 4.2 (Barrett et al., 2005). A total of 10,000 permutations were used to assess the p-value of the haplotype using the build in function of Haploview 4.2.

All variations within the exons genes were tested with the adaptive sum test of rare and common variant effects, as implemented in the Sequence Kernel Association Test (SKAT) (Ionita-Laza et al., 2013, Wu et al., 2011). Tests were performed with setting the minor allele frequency (MAF) for rare variants on 0.05, 0.01 and the default setting. Bonferroni-correction and False Discovery Rate (FDR) correction for 46 tests, equal to the number of included genes, as implemented in the R package, were applied for each SKAT generated p-value (R, 2008).

p-Values between marker annotations of healthy controls, pneumococcal meningitis patients and outcome were tested with the R built in Chi-square test (R, 2008).

Cytokines level differences between genotypes were compared using a Mann-Whitney *U* test.

## 3 Results

### 3.1 Clinical Characteristics of Pneumococcal Meningitis Patients

In this study we included 435 controls and 416 pneumococcal meningitis patients. Median age of included patients was 61 years (interquartile range 50–69) and 216 were female (50%). Upon discharge 146 (34%) had an unfavorable outcome (GOS 1 to 4) and 34 (8%) died.

### 3.2 Genetic Variation

Sequencing the exomes and flanking regions of 46 innate immune genes of 851 individuals resulted in 2099 variations after quality control. The majority of variations had a MAF below 0.01 (80%), indicating that the variation within the innate immune genes had a low prevalence within the cohort. A total of 930 variations (44.3%) were found in only one individual. The total presence of unique variations did not differ between meningitis patients and controls.

Nonsense and frame-shift mutations leading to a premature stop could lead to non-functional proteins of the transcript. Within our individuals 12 variations were found that caused a stop in a total of 10 innate immune proteins (see Table 1). Compared to all variations, there was no overrepresentation of stop-gained variations between patients and healthy controls (p = 0.34) or between patients with favorable and unfavorable outcome (p = 0.25). In relation to disease susceptibility and outcome, one heterozygous stop-gained variation in the IRAK 4 gene was observed in 4 patients who mainly had a favorable outcome (Table 1). Three other stop-gained variations were found only within the group of patients with an unfavorable outcome and were located in the *CXCL8, TLR2* and *TIRAP* genes (Table 1).

**Fig. 1:**
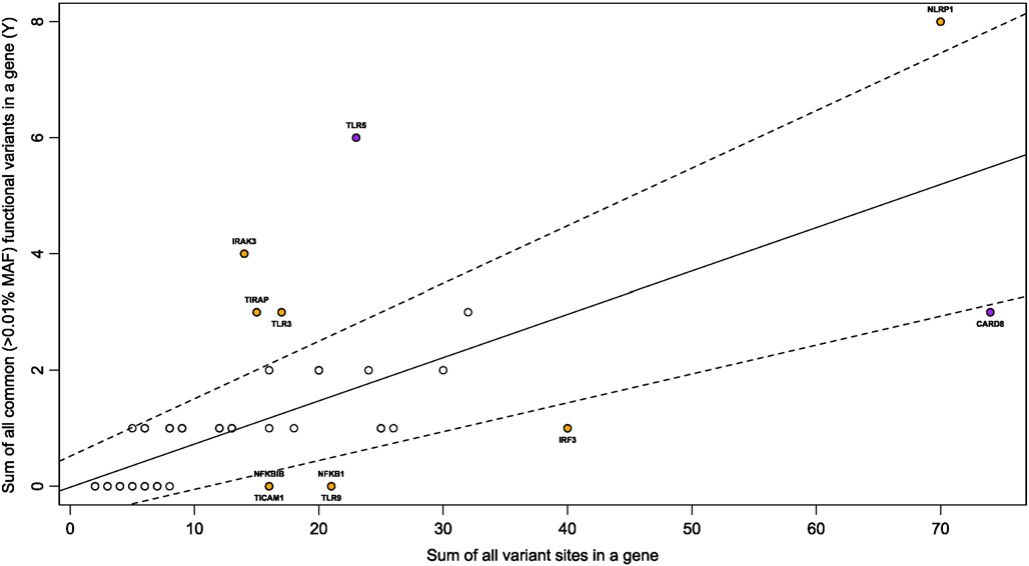
Regression plot illustrating the regression of the sum of all common functional variants on all variants. Dashed lines indicate the 95% CI. Samples outside the 95% CI have been colored orange. Common has been set on a MAF of 1%. Most common functional variant tolerated (TLR5) and intolerant (CARD8) genes are colored purple.

Genes can have more functional genetic variation altering the function of the protein. To evaluate the included 46 innate immune genes we calculated the RVIS. The RVIS indicates the extent to which a gene number of common functional mutations departure from genes with a similar amount of mutations (Petrovski et al., 2013). Most common functional variation was found in TLR5 and CARD8 had the least common functional variants (Fig. 1). When comparing distribution of functional to all non-functional variants between patients and controls or patients disease outcome we observed no differences (p = 0.75 and p = 0.37).

### 3.3 Pneumococcal Meningitis Susceptibility and Outcome Associated Innate Immune Markers

Results of the association study on the innate immune genetic variation with susceptibility are shown in Table 2. Association was first tested with the fisher exact test because of the high number of variants with a low MAF. The strongest signal was a T-variant in an intron of *CARD8* (rs2008521), showing a higher frequency in cases compared to the controls (OR 1.82, CI 1.28–2.75, p = 8.2e − 04). Preforming a logistic regression adjusted for population stratification and age resulted in a p-value of 6.1e − 4 (OR 1.93, CI 1.33–2.81) for the *CARD8* rs2008521 variation. With the regression analysis a stronger signal is an A-variant located in the intron of *CXCL1* (rs56078309) (OR 1.96, CI 1.34–2.87, p = 8.2e − 04). However, after correction for multiple testing, no associations reached significance.

Haplotype associations for each gene showed no strong association signals for pneumococcal susceptibility. Testing the genes for total variation differences, with SKAT, based on all variations showed *TLR5* (p = 0.03) as the strongest association and *TLR3* (p = 0.03) when weight on common and rare variations. With correction for multiple testing these signals did not reach significance.

Differences between allele frequencies within patients grouped on outcome are shown in Table 3. The strongest signal, standing out compared to the other signals, was a G-variant in the 3-prime UTR region of *IRAK4* (rs4251552) showing a higher frequency in patients with an unfavorable outcome (OR 2.86, CI 1.58–5.18, p = 4.8e − 04). Adjusting for population stratification, age and immunosuppression of the patients resulted in a p-value of 6.7e − 04 (OR3.00, CI 1.59–5.66). When applying this correction the strongest signal was a synonymous G-variation in *NOD2* (rs2067085) (OR 2.16, CI 1.40–3.34, p = 5.1e − 04). After correcting for multiple testing the associations were no longer significant.

**Fig. 2:**
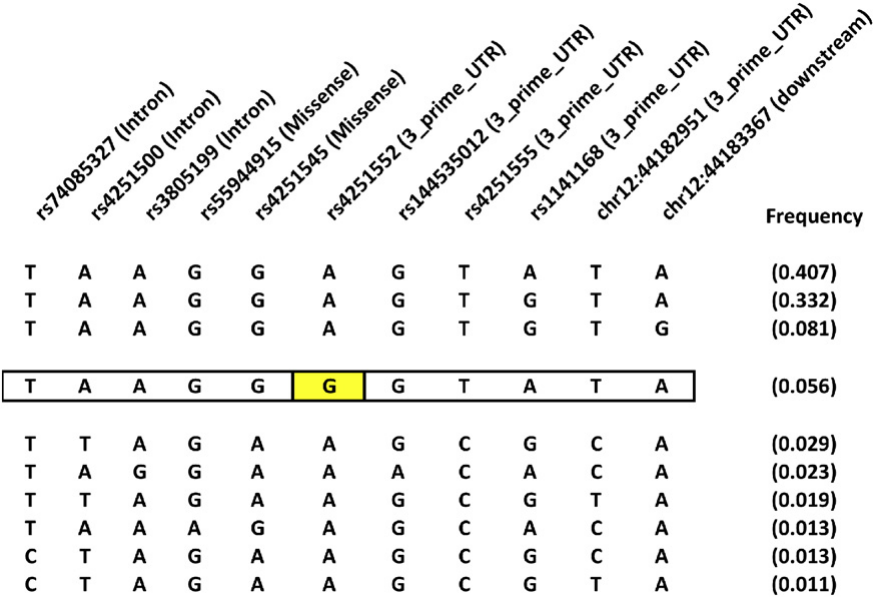
*IRAK4* haplotypes with a frequency of at least 1%. Significant *IRAK4* haplotype (p = 4.0e − 04, after 10,000 permutation test p = 0.004) is outlined and the rs4251552 is highlighted in yellow. Above all SNPs are the rs numbers and between parenthesis the functional annotation. Frequency of the haplotype was 0.095 in patients with unfavorable and 0.036 within the favorable group.

Haplotype association for *IRAK4* showed a significant association, after correction for multiple testing, of one haplotype (p = 4.0e − 04, after 10,000 permutation test p = 0.004). This association is driven by the *IRAK4* rs4251552 variation, which was one of the strongest signals in the single variation analyses (Fig. 2).

SKAT analysis based on all variations showed that *PARP1* (p = 0.06) as the strongest association and *TICAM1* (p = 0.03) when weight on common and rare variations. With correction for multiple testing these signals did not reach significance.

### 3.4 Effect of the Genetic Associations on CSF Cytokine Concentrations

**Fig. 3:**
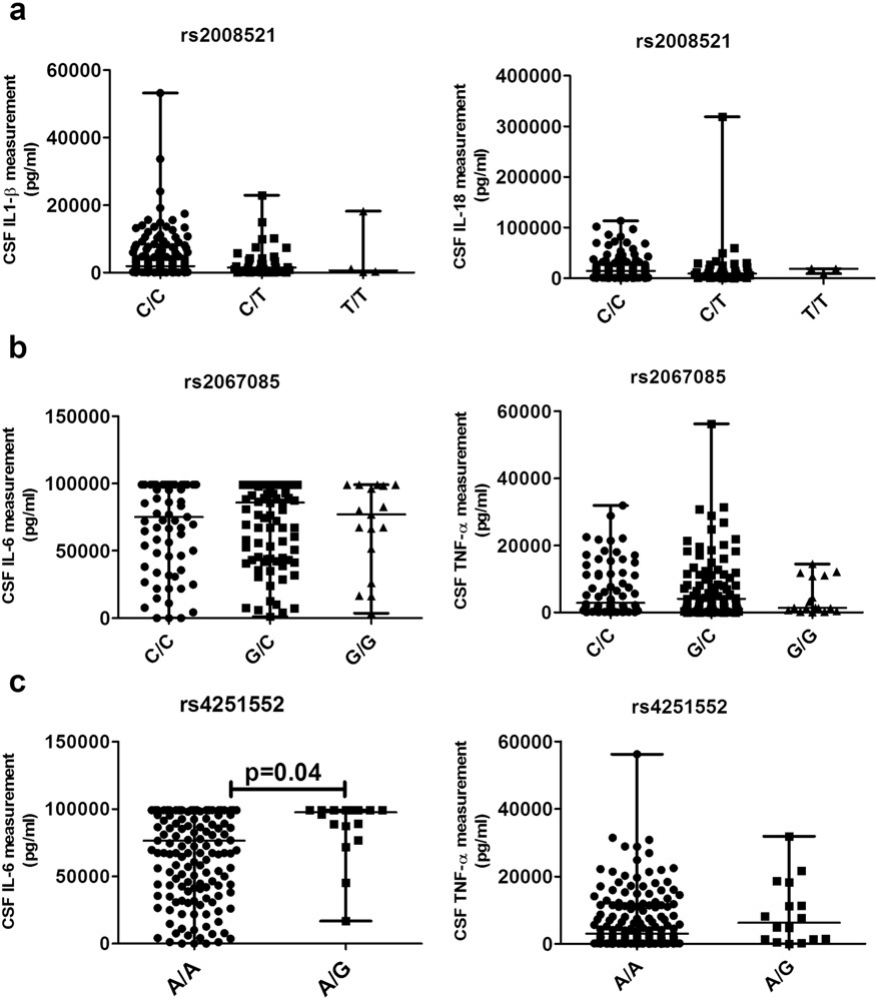
CSF protein cytokine levels of the *CARD8* rs2043211 *NOD2* rs 2067085 and *IRAK4* rs4251552 genotypes. Cytokines IL1-β, IL-18, IL-6 and TNF-α were determined within the CSF from the diagnostic lumbar puncture samples taken from the patients. (a) Patients grouped on the *CARD8* rs2043211 CC, TC and TT genotypes. For IL1-β there were 133 CC, 35 TC and 4 TT patients and 131 CC, 35 TC and 3 TT for IL-18. (b) Patients grouped on their *NOD2* rs206085 CC, CG and GG genotypes. For IL-6 and TNF-α 67 CC, 87 GC and 17 GG were included. (c) Patients grouped on their *IRAK4* rs4251552 AA and AG genotypes. IL-6 includes 159 AA and 16 AG patients and for TNF-α it were 158 AA and 16 AG patients. All data are summarized as median and differences were tested using the Mann-Whitney *U* test.

From a subset of the patients we have measured the cytokines IL-6, TNF-α, IL1-β and IL-18. For *CARD8*, *NOD2* and *IRAK4* it is known that deficiencies in the gene alter the expression of some of these cytokines (Ko et al., 2009, Ku et al., 2007, Ogura et al., 2001). *CARD8* is a caspase recruitment domain-containing protein involved in activation of caspases leading to IL1-β and IL-18 cytokine production (Razmara et al., 2002). IL1-β and IL-18 CSF measurements from pneumococcal meningitis patients showed no altered cytokine responses in different genotype groups (Fig. 3a). *NOD2* is involved in the recognition and innate immune response against *S. pneumoniae* activating inflammatory cytokines, like IL-6 and TNF-α through NF-κB activation (Opitz et al., 2004, Strober et al., 2006). The associated NOD2 variation showed no difference in cytokine CSF measurements grouped on their genotypes (Fig. 3b). IRAK4 is the initial kinase near TLR receptors to activate downstream effectors, such as the cytokines IL-6 and TNF-α, we studied these protein levels in relation with genotype (Edwards and Chang, 1975). Cytokine measurements from CSF in 175 patients showed that individuals with AG or GG genotype had significantly higher levels of IL-6 compared to individuals with AA genotype (median AA 75 ng/ml interquartile range (IQR) 42–99 vs. AG/GG 98 (IQR 85–99), p = 0.04, Fig. 3c). No differences in TNF-α were observed.

### 3.5 Evaluating Known Invasive Pneumococcal Disease and Meningitis Genetic Associations

Table 4 summarizes all genetic variations that we have sequenced and is reported to be associated with either invasive pneumococcal disease (IPD) or meningitis. From the genes *CARD8*, IL-10, IRAK4, MAL, MYD88, TLR2 and TLR4 a total of 10 variations could be replicated. None of the variations showed a significant association with pneumococcal meningitis susceptibility or outcome.

## 4 Discussion

This study used full sequencing of the coding region and flanking intron boundaries of 46 innate immune genes from pneumococcal meningitis patients and healthy controls to search for genetic association with susceptibility and disease outcome. We identified variations in *CXCL1* (rs56078309) and *CARD8* (rs2008521) as the strongest signals associated with susceptibility. Variations within the *NOD2* (rs2067085) and *IRAK4* (rs4251552) were associated with pneumococcal meningitis disease outcome. Within the *IRAK4* gene the found association rs4251552 defined a haplotype that is significantly associated with pneumococcal disease outcome. Furthermore, pneumococcal meningitis patients with the *IRAK4* risk allele had higher levels of IL-6 in their CSF with the diagnostic lumbar puncture, suggesting that these patients have an increased pro-inflammatory response during their disease.

*IRAK4* is a downstream TLR signaling protein and deficiency is a known cause of recurrent pneumococcal disease (Picard et al., 2003, Picard et al., 2010). *IRAK4* deficient PBMCs of patients have altered transcription and impaired cytokine response after being activated with various TLR agonists and pathogens (Alsina et al., 2014, Ku et al., 2007). In our four patients bearing a heterozygous stop-gained *IRAK4* mutation we did not find any specific clinical characteristics. Deficiency of *IRAK4* leads to dysfunctional TLR signaling and increased susceptibility. In contrast the observed increased IL-6 trend with the *IRAK4* associated rs4251552 displays the opposite picture with a stronger pro-inflammatory response, which has been shown to be harmful for meningitis outcome (Fig. 3) (Mook-Kanamori et al., 2011). The central role of the *IRAK4* gene during IPD makes it a plausible candidate for adjuvant therapies. Our findings indicate that these therapies have to focus on reducing the *IRAK4* expression, or protein quantity, during the infection to lower the inflammatory response.

Sequencing of 46 innate immune genes exomes all involved in pneumococcal immune response did not result in many strong signals. After correction for multiple testing none of the signals reached significance (Table 2, Table 3). Because of the uniqueness of our cohort we could not replication our strongest signals in another pneumococcal meningitis cohort. With this study we were able to look at the signals of variations previously associated IPD and pneumococcal meningitis. Variations for *CARD8*, *IL-10*, *IRAK4*, *MAL*, *MYD88*, *TLR2* and *TLR4* genes have been reported in association studies and were included in our data (Carrasco-Colom et al., 2015, Ellis et al., 2015, Geldhoff et al., 2013, Khor et al., 2007, Schaaf et al., 2003, van Well et al., 2013, van Well et al., 2012, Yuan et al., 2008). None of the published variants reach significance within our analysis, even prior to correction for multiple testing (Table 4). Of these variations those in *CARD8*, *TLR2* and *TLR4* were reported to be associated with bacterial meningitis (Geldhoff et al., 2013, van Well et al., 2013, van Well et al., 2012). Lack of association could be the result of the number of patients included in the studies or grouping of different causal bacteria from the patients. The lack of association for *MYD88*, *IRAK4* and *IKBKG* is in line with the invasive pneumococcal disease results of Ellis et al. (Ellis et al., 2015).

Neither single SNPs, haplotype nor burden analysis could show strong associations. The lack of associations with variation in innate immunity genes, a known pathway involved in our defense against infection, could be the result of the evolutionary pressure on the innate immune system. The innate immune system genes are subjected to purifying selection (Ferrer-Admetlla et al., 2008). Purifying selection is the selective removal of alleles that are harmful because they alter the protein function significantly (Karlsson et al., 2014). The finding of a higher frequency of missense variations with a MAF < 0.01 could be the result of this purifying selection that prevents them from gaining higher frequency in the population. Despite the purifying selection it has been shown that cell-surface expressed TLRs show relaxation of the selective constraints leading to the increased presence of missense and stop-gain mutations (Barreiro et al., 2009). In line with these relaxations we observed that most stop-gained variations are located within TLRs, like the *TLR5* (rs5744168), which can have high frequency within the population (Table 1). The high frequency of rs5744168 has already been reported and has been associated with altered susceptibility to Legionnaires disease (Hawn et al., 2003). High RVIS of the *TLR5* gene is indicating that it contains the most functional common variations of all included genes is in line with the relaxation of the selective constraints (Fig. 1). For all other stop-gained variations we only found them at low frequency and in heterozygote state making it difficult to evaluate their effect on susceptibility and outcome.

Our study focuses mainly on the variation located within the protein coding regions and misses most of the genetic variations near the genes involved in transcription regulation. Association studies in IPD also reported variations in the nuclear factor of kappa light polypeptide gene enhancer in B-cell inhibitors alpha (*NFKBIA*) and (*NFKBIE*) epsilon, Toll-interleukin 1 receptor (*TIRAP*), interleukin cytokines *IL-6*, *IL-10* and a macrophage migration inhibitory factor (*MIF*) microsatellite (Chapman et al., 2007, Martin-Loeches et al., 2012, Schaaf et al., 2005, Schaaf et al., 2003). All these variations are located upstream of the genes and affect transcription and are not captured during exon sequencing, which is a limitation of our study.

A further limitation in our analysis on genetic variation and outcome of disease is that treatment modalities varied per patient. The study is observational and choice of treatment was at the discretion of the treating physician. Because of the multitude of antibiotic regimens we were unable to apply a correction for this. However, we think it is unlikely this has a major influence on our results.

The number of individuals included in this study is the largest pneumococcal meningitis cohort at this time. Results did not show many strong associations, with high frequencies, that could be used as pneumococcal meningitis risk markers for disease onset or progress. But the association results do show which innate immune genes affected by variation influence pneumococcal meningitis and point to import hubs for treatment during the disease. The low frequency of most variations within the cohort makes it hard to detect associations. This was not restricted to the single variant analysis, as the gene based mutation burden analysis by SKAT also found no associations, when corrected for multiple testing. Increasing the cohort size and replication of the most significant variants detected will eventually show whether these rare variants contribute to pneumococcal meningitis susceptibility and outcome.

*CXCL1* (rs56078309) and *CARD8* (rs2008521) variations were the strongest associations for susceptibility. For disease outcome this were the *NOD2* (rs2067085) and *IRAK4* (rs4251552) variations. For the *IRAK4* rs4251552 variation a trend of increased CSF IL-6 was observed for patients with the risk allele. Because of the dominant role in the innate immune signaling the finding that *IRAK4* influences outcome makes it a candidate for adjuvant therapies.

The following is the supplementary data related to this article.

Supplement Table 1 List of 46 exome sequenced innate immunity genes. Supplement Table 1.

## Conflict of Interest

The authors declare that they have no conflict of interest.

## Author Contributions

The manuscript was drafted by B.F., M.C.B, D.v.d.B. A.v.d.E, M.C.B, D.v.d.B. were involved in the data collection. Genetic data sequencing, analyzing and interpretation of the data was performed by B.F, A.J, A.H.Z, F.B. M.V.S, M. G. and B.F measured and analyzed the human cytokine data. All authors discussed, read and approved the final version of the manuscript.

## Figures and Tables

**Table 1:** STOP-Gained variations.

Chr	Location	Gene	rs	Codon	Healthy controls	Meningitis patients	Favorable outcome	Unfavorable outcome
Stop-gained variations								
1	223285200	*TLR5*	rs5744168	Arg/*	4 homozypotes 49 heterozygotes	2 homozypote 52 heterozygotes	2 homozypote 36 heterozygotes	16 heterozygotes
2	113888645	*IL1RN*	rs121913161	Glu/*	1 heterozygote			
3	38181977	*MYD88*	–	Arg/*	1 heterozygote			
4	74607285	*CXCL8*	rs188378669	Glu/*		1 heterozygote		1 heterozygote
4	154625126	*TLR2*	rs146476103	Ser/*		1 heterozygote		1 heterozygote
4	154625398	*TLR2*	rs62323857	Arg/*	3 heterozygotes	2 heterozygotes	1 heterozygotes	1 heterozygote
9	120475449	*TLR4*	–	Leu/*	1 heterozygote	4 heterozygotes	3 heterozygotes	1 heterozygote
9	120476185	*TLR4*	rs5030720		1 heterozygote			
11	126162878	*TIRAP*	rs149490135	Arg/*		1 heterozygote		1 heterozygote
12	44172041	*IRAK4*	rs121908002	Gln/*		4 heterozygotes	3 heterozygotes	1 heterozygote
12	104333351	*HSP90B1*	–	Ser/*	6 heterozygotes	4 heterozygotes	2 heterozygotes	2 heterozygotes
19	10394174	*ICAM1*	–	Glu/*	5 heterozygotes	5 heterozygotes	2 heterozygotes	3 heterozygotes

**Table 2:** Top associations of sequenced variants with susceptibility to pneumococcal meningitis.

Chr	Location^a^	rs	Function^b^	Gene	Alleles		A1 frequency		p-Value		OR (± 95 CI)
A1	A2	Cases	Control	Unadjusted	FDR^c^
Susceptibility (Five highest Fisher exact test signals)											
19	48715269	rs2008521,	Intron	*CARD8*	T	C	0.115	0.067	8.2e − 4	1	1.82 (1.28–2.57)
19	48711789	rs1968441	3_prime_UTR	*CARD8*	T	G	0.112	0.067	0.002	1	1.76 (1.24–2.48)
19	48711902	rs1968440	3_prime_UTR	*CARD8*	A	G	0.112	0.068	0.002	1	1.74 (1.23–2.46)
11	36510096	rs5030481	3_prime_UTR	*TRAF6*	C	T	0.029	0.009	0.003	1	3.44 (1.47–8.03)
4	74736180	rs56078309	Intron	*CXCL1*	A	G	0.112	0.070	0.003	1	1.68 (1.19–2.35)

Susceptibility (five highest logistic regression test signals^d^)											
4	74736180	rs56078309	Intron	*CXCL1*	A	G	0.112	0.070	4.8e − 4	0.9	1.96 (1.34–2.87)
19	48715269	rs2008521,	Intron	*CARD8*	T	C	0.115	0.067	6.1e − 4	1	1.93 (1.33–2.81)
19	48711789	rs1968441	3_prime_UTR	*CARD8*	T	G	0.112	0.067	0.001	1	1.86 (1.28–2.70)
19	48711902	rs1968440	3_prime_UTR	*CARD8*	A	G	0.112	0.068	0.002	1	1.83 (1.26–2.65)
11	36510096	rs5030481	3_prime_UTR	*TRAF6*	C	T	0.029	0.009	0.002	1	4.69 (1.75–12.6)

a Location is based on GRCh37/hg19.

b The functional consequence of the variation or region of the gene transcript it is located.

c Adjusted p-value after False Discovery Rate (FDR) correction for type I errors.

d Analysis include the first ten MDS and Age as covariates.

**Table 3:** Top associations of sequenced variants with outcome of pneumococcal meningitis

Chr	Location^a^	rs	Function^b^	Gene	Alleles		A1 frequency		p-Value		OR (± 95 CI)
A1	A2	Unfavorable	Favorable	Unadjusted	FDR^c^
Disease outcome (five highest Fisher exact test signals)											
12	44181141	rs4251552	3_prime_UTR	*IRAK4*	G	A	0.095	0.035	4.8e − 04	0.68	2.86 (1.58–5.18)
4	103497947	–	Intron	*NFKB1*	T	A	0.069	0.025	0.003	1	2.91 (1.45–5.82)
11	36509357	rs5030508	3_prime_UTR	*TRAF6*	T	C	0.031	0.083	0.003	1	0.35 (0.17–0.72)
4	103497946	–	Intron	*NFKB1*	A	T	0.066	0.025	0.005	1	2.77 (1.37–5.60)
22	22142954	rs56080243	Intron	*MAPK1*	T	C	0.024	0.003	0.009	1	6.88 (1.42–33.32)

Disease outcome (Five highest logistic regression test signals^d^)											
16	50733859	rs2067085	Synonymous	*NOD2*	G	A	0.406	0.345	5.1e − 04	0.71	2.16 (1.40–3.34)
12	44181141	rs4251552	3_prime_UTR	*IRAK4*	G	A	0.095	0.035	6.7e − 04	0.92	3.00 (1.59–5.66)
4	103497947	–	Intron	*NFKB1*	T	A	0.069	0.025	0.003	1	3.07 (1.45–6.50)
11	36509357	rs5030508	3_prime_UTR	*TRAF6*	T	C	0.031	0.083	0.004	1	0.33 (0.16–0.71)
4	103497946	–	Intron	*NFKB1*	A	T	0.066	0.025	0.005	1	2.99 (1.39–6.40)

a Location is based on GRCh37/hg19.

b The functional consequence of the variation or region of the gene transcript it is located.

c Adjusted p-value after False Discovery Rate (FDR) correction for type I errors.

d Analysis include the first ten MDS, Age and immunosuppressed status as covariates.

**Table 4:** Validation of genetic variants previously reported in invasive pneumococcal disease in the current pneumococcal meningitis cohort.

Chr	Location^a^	rs^b^	Function^c^	Gene	p-Value	OR (± 95 CI)	p-Value	OR (± 95 CI)
Case/control	Outcome
Signals of reported IPD and meningitis associations								
19	48737706	rs2043211	Missense	*CARD8*^d^	0.98	1.01 (0.68–1.48)	0.09	1.65 (0.93–2.94)
1	206941864	rs3024496^e^	3_prime	*IL-10*	0.31	1.18 (0.86–1.63)	0.47	1.19 (0.74–1.91)
12	44172041	rs121908002	Stop gained	*IRAK4*	0.12^f^	–	1^f^	0.64 (0.07–6.19)
12	44177511	rs55944915	Missense	*IRAK4*	0.67	1.21 (0.50–2.92)	0.99	0.99 (0.28–3.56)
12	44180295	rs4251545	Missense	*IRAK4*	0.11	1.33 (0.93–1.89)	0.38	1.26 (0.76–2.08)
11	126162843	rs8177374	Missense	*MAL*	0.10	1.28 (0.95–1.72)	0.30	1.30 (0.86–1.98)
3	38184370	rs6853	3_prime	*MYD88*	0.96	1.01 (0.74–1.37)	0.93	0.98 (0.62–1.55)
4	154626317	rs5743708	Missense	*TLR2*	0.99	0.99 (0.62–1.62)	0.93	0.97 (0.48–1.98)
9	120475302	rs4986790	Missense	*TLR4*	0.47	1.16 (0.78–1.72)	0.58	1.17 (0.67–2.03)
9	120475602	rs4986791	Missense	*TLR4*	0.45	1.17 (0.78–1.76)	0.99	1.00 (0.56–1.79)

a Location is based on GRCh37/hg19.

b Variations have been reported in Carrasco-Colom et al. (2015), Ellis et al. (2015), van Well et al. (2012) and Yuan et al. (2008).

c The functional consequence of the variation or region of the gene transcript it is located.

d Additive model p-value = 0.5.

e SNP in linkage with the associated variation rs1800896 (upstream_gene_variant).

f Due to low frequency and only found in cases the Fisher exact test was used.
