# Supplementary material for: Variation of 46 Innate Immune Genes Evaluated for their Contribution in Pneumococcal Meningitis Susceptibility and Outcome
Source: eBioMedicine. 2016 Jul 12;10:77–84. doi: 10.1016/j.ebiom.2016.07.011 (PMC5006661; doi:10.1016/j.ebiom.2016.07.011)
Supplement: Supplement Table 1 — List of 46 exome sequenced innate immunity genes. [file mmc1.docx]

**Supplement**

**Supplementary table 1**

| **46 Exome sequenced innate immunity genes** |
| --- |
| Bruton agammaglobulinemia tyrosine ***(BTK)*** |
| Caspase recruitment domain family, member 8 ***(CARD8)*** |
| Caspase recruitment domain family, member 9 ***(CARD9)*** |
| Caspase 1, apoptosis-related cysteine peptidase ***(CASP1)*** |
| CD14 molecule ***(CD14)*** |
| Chemokine (C-X-C motif) ligand 1 ***(CXCL1)*** |
| *Chemokine (C-X-C motif) ligand 2* ***(CXCL2)*** |
| *Chemokine (C-X-C motif) ligand 8* ***(CXCL8)/(IL-8)*** |
| Heat shock protein 90kDa beta (Grp94), member 1 ***(HSP90B1)*** |
| Intercellular adhesion molecule 1 ***(ICAM1)*** |
| Interferon, gamma ***(IFNG)*** |
| Inhibitor of kappa light polypeptide gene enhancer in B-cells, kinase gamma ***(IKBKG)/(NEMO)*** |
| Interleukin 10 ***(IL-10)*** |
| Interleukin 12B ***(IL-12B)*** |
| Interleukin 1 Beta ***(IL1-Β)*** |
| Interleukin 1 receptor antagonist ***(IL1RN)*** |
| Interleukin 6 ***(IL-6)*** |
| Interleukin-1 receptor-associated kinase 1 ***(IRAK1)*** |
| Interleukin-1 receptor-associated kinase 3 ***(IRAK3)*** |
| Interleukin-1 receptor-associated kinase 4 ***(IRAK4)*** |
| Interferon regulatory factor 3 ***(IRF3)*** |
| Mitogen-activated protein kinase 1 ***(MAPK1)*** |
| Macrophage migration inhibitory factor ***(MIF)*** |
| Myeloid differentiation primary response gene 88 ***(MYD88)*** |
| Nuclear factor of kappa light polypeptide gene enhancer in B-cells 1 ***(NFKB1)*** |
| Nuclear factor of kappa light polypeptide gene enhancer in B-cells 2 (p49/p100) ***(NFKB2)*** |
| Nuclear factor of kappa light polypeptide gene enhancer in B-cells inhibitor, alpha ***(NFKBIA)*** |
| Nuclear factor of kappa light polypeptide gene enhancer in B-cells inhibitor, beta ***(NFKBIB)*** |
| NLR family, pyrin domain containing 1 ***(NLRP1)*** |
| NLR family, pyrin domain containing 3 ***(NLRP3)*** |
| Nucleotide-binding oligomerization domain containing 2 ***(NOD2)*** |
| Poly (ADP-ribose) polymerase 1 ***(PARP1)*** |
| PYD and CARD domain containing ***(PYCARD)*** |
| Toll-like receptor adaptor molecule 1 ***(TICAM1)*** |
| Toll-like receptor adaptor molecule 2 ***(TICAM2)*** |
| Toll-interleukin 1 receptor (TIR) domain containing adaptor protein ***(TIRAP)/(MAL)*** |
| Toll-like receptor 2 ***(TLR2)*** |
| Toll-like receptor 3 ***(TLR3)*** |
| Toll-like receptor 4 ***(TLR4)*** |
| Toll-like receptor 5 ***(TLR5)*** |
| Toll-like receptor 9 ***(TLR9)*** |
| Tumor necrosis factor ***(TNF)*** |
| Tumor necrosis factor, alpha-induced protein 3 ***(TNFΑIP3)*** |
| TNF receptor-associated factor 6, E3 ubiquitin protein ligase ***(TRAF6)*** |
| Unc-93 homolog B1 ***(UNC93B1)*** |
| Vascular cell adhesion protein 1 ***(VCAM1)*** |
